# Geographical Limits of the Southeastern Distribution of *Aedes aegypti* (Diptera, Culicidae) in Argentina

**DOI:** 10.1371/journal.pntd.0001963

**Published:** 2013-01-31

**Authors:** Leonardo M. Díaz-Nieto, Arnaldo Maciá, M. Alejandra Perotti, Corina M. Berón

**Affiliations:** 1 Centro de Estudios de Biodiversidad y Biotecnología (CEBB-CIB-FIBA-Mar del Plata) – CONICET, Mar del Plata, Argentina; 2 División Entomología, Facultad de Ciencias Naturales y Museo de La Plata (UNLP), La Plata, Argentina; 3 School of Biological Sciences, University of Reading, Reading, United Kingdom; Monash University, Australia

## Background

*Aedes aegypti* (Linnaeus) is a human-biting mosquito and the primary vector of human dengue and yellow fever viruses; it is also considered the principal vector of Chikungunya virus in Asia [1], [2]. In particular, dengue and dengue hemorrhagic fever constitute an important burden to humankind in terms of morbidity and mortality. About 3.6 billion people in the tropics, mainly in Asia, the Western Pacific region, the Caribbean, and Central and South America, live under risk of infection with one or more of the four dengue virus serotypes (DEN-1 to DEN-4), and recent reports estimate over 230 million infections, over 2 million cases of the severe form of the disease, and 21,000 deaths [3].

It is believed that *A. aegypti* originally migrated from West Africa to the North and South America in the 15th century aboard slave ships, after which yellow fever appeared in the New World. Presumably the yellow fever virus was introduced by travellers on these ships, especially African slaves. The adaptation of this insect to survive in human environments was crucial for colonization and development in water storage containers in the holds of sailing ships [4]. At present, *A. aegypti* lives in close proximity to people, in urban areas, breeding in all types of domestic and peridomestic collections of fresh water, including flower vases, water drums, tins, broken coconut shells, old tires, and gutters. A major range of expansion of *Aedes* mosquitoes into these urban areas is also attributable to the adaptation of the genera *Aedes* to breed in water-holding automobile tires [5].

*A. aegypti* is a tropical and subtropical species spanning a geographical distribution from 35°N to 35°S. Its lower thermal threshold corresponds to 10°C isotherms during the winter, and although it has been found up to 45°N, its presence in colder regions is due to its ability to colonize new areas during the warm season [6]. In South America, the historic direction of dispersal of *Aedes* mosquitoes has been towards higher latitudes and from tropical to sub-tropical areas, in particular in the Southern Cone. We propose that the southeastern movement of *A. aegypti* might be related to human migrations from rural areas to towns lacking in a proper housing policy and essential services like water, and sewage disposal systems (http://www.migraciones.gov.ar/pdf_varios/estadisticas/Patria_Grande.pdf) [7].

**Figure 1:**
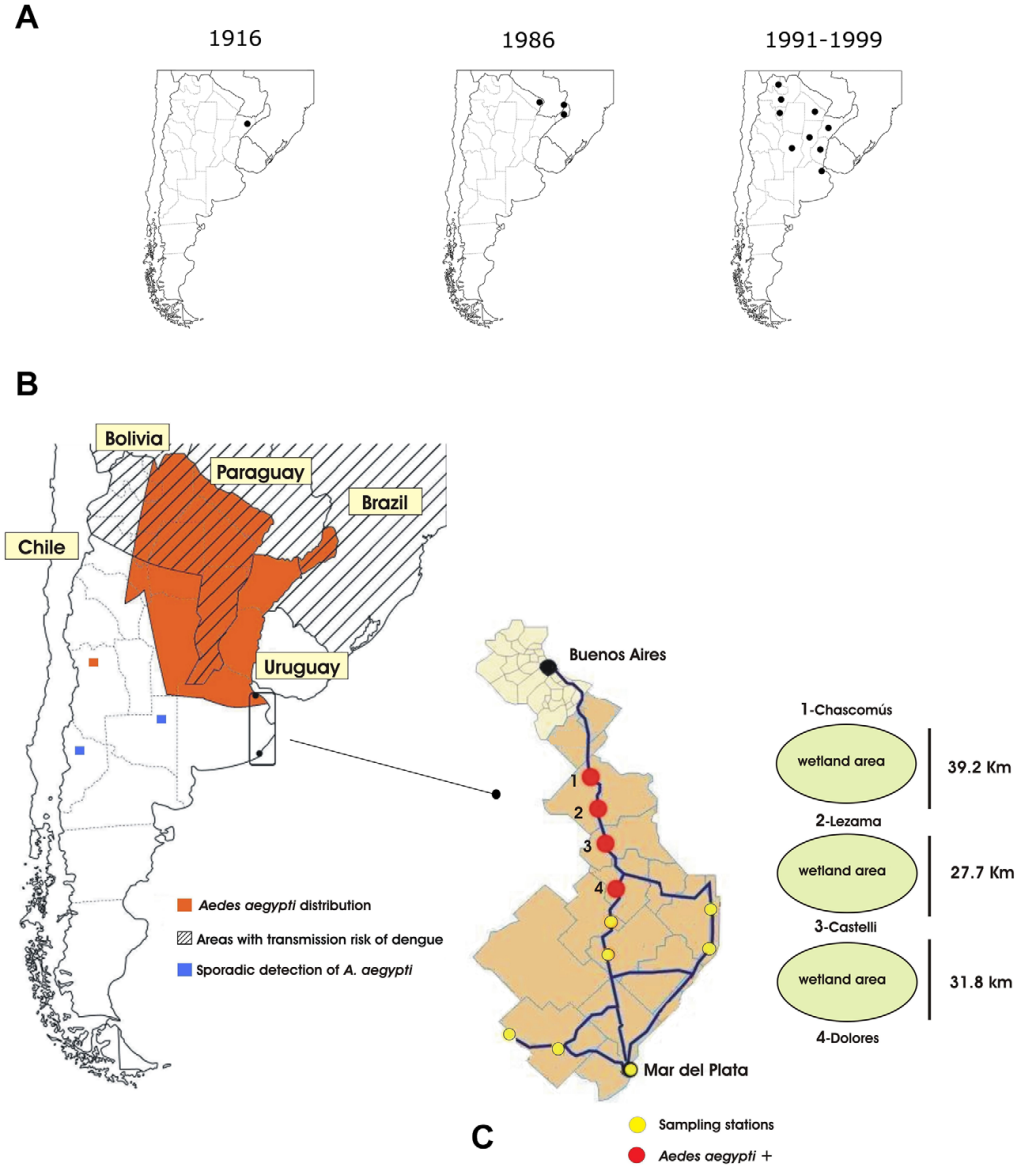
*Aedes aegypti* and dengue fever in South America. (**A**) Historical distribution of *A. aegypti* in Argentina, indicating 1916, the first dengue-like epidemic; 1986, re-infestation locations; and biogeographical records between 1991 and 1999. (**B**) Current geographic distribution of *A. aegypti* and regions with risk of transmission of dengue in South America. (**C**) Studied area, showing highways between Buenos Aires and Mar del Plata cities, sampling points, and distances between them. (A and B) adapted from Curto et al., Vezzani and Carbajo [10], [15], and http://www.healthmap.org/dengue/index.php.

Between the 1950s, 1960s, and most of the 1970s, epidemic dengue was rare in Central and South America because *A. aegypti* had been eliminated from most of the countries. The eradication program organized by the Pan American Health Organization (PAHO) was discontinued in the early 1970s, and consequently the mosquito was reintroduced in countries from which it had been eradicated [6], [8]. In Argentina, the earliest records of *A. aegypti* go back to the 1900s and are concurrent with the dengue-like epidemic of 1916, which affected the coastal areas of the Uruguay River (31°44′S, 60°31′W) [9]. However, in 1986 re-infestation took place along the northern border with Paraguay, spreading over wide areas of the country. Nowadays, the current geographical distribution of *A. aegypti* in Argentina is wider than during its eradication in 1967 [10], [11]. Recently it has been demonstrated that the three *A. aegypti* main haplogroups identified in Argentina represent different colonization events, probably from neighboring countries: Bolivia, Paraguay, and Brazil (Figure 1A and 1B) [7]. Particularly, in Buenos Aires Province, the most densely populated area of the country, the records of high abundances of well-established populations of *A. aegypti* were taken in La Plata (capital of the province) and in Buenos Aires (capital city of the country), both located on the east coast, and the southernmost findings were recorded in Chascomús, 132 km from Buenos Aires city (35°33′S, 58°00′W, Figure 1) [10]–[15].

On the other hand, cases of dengue have increased in the last few years in Argentina. From January to June 2012, 2,043 patients with symptoms were reported, and 194 were confirmed with serotypes DEN-1, DEN-2, or DEN-3 (http://www.msal.gov.ar/dengue/images/stories/partes_dengue/parte74.pdf). In 2011 PAHO released an epidemiological alert due to the introduction of DEN-4 serotype in the Americas (http://new.paho.org), with Brazil, Paraguay, and Bolivia countries at high risk of dengue infection with 57,267 possible cases and 5 deaths (Brazil); 10,827 suspected cases and 30 deaths (Paraguay); and 3,233 notified cases with 28 deaths (Bolivia) (Figure 1B) (http://www.msal.gov.ar/dengue/images/stories/partes_dengue/parte74.pdf).

In the United States, the dispersal of *Aedes albopictus* Skuse offered an opportunity to understand the synanthropic behavior of *Aedes* mosquitoes. The mosquito was introduced in 1985 in the continental territory through shipments of used tires from Asia that contained eggs [16]. In subsequent years, the pattern of spread of this container-dwelling species followed the main interstate highways [17], quickly reaching and colonizing several new areas of the US in a few years. We wondered whether *A. aegypti* would present a similar behavior, and is making use of human transportation [18]. For this, we investigated the occurrence of the mosquito in major roads connecting densely populated cities with the southeast of Argentina (Table 1).

**Table 1:** Characteristics of cities connected by Route N° 2 in Buenos Aires Province (http://www.censo2010.indec.gov.ar/).

City	Area (km^2^)	Population Size	Number of Households
Buenos Aires	2,681	12,801,365	3,147,638
Chascomús	3,452	38,477	18,277
Lezama	1,102	4,111	Nd^b^
Castelli	2,063	8,206	3,448
Dolores	1,973	26,601	10,687
General Guido	2,814	2,814	1,508
Maipú	2,641	10,172	4,375
Mar del Plata	1,461	618,989	308,570
MdP, summer time^a^	1,461	2,000,000	Nd^b^

a MdP, Mar del Plata.

b No data.

**Figure 2:**
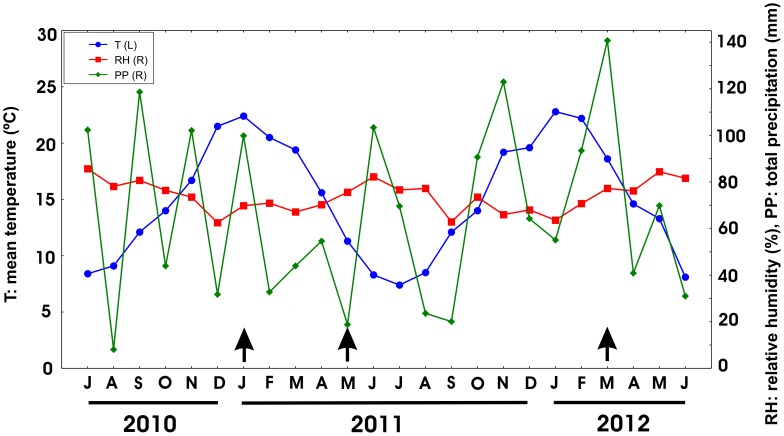
Weather conditions of the studied area, from July 2010 to June 2012. On the left mean temperature in °C (T), on the right % of relative humidity (RH) and total precipitation in mm (PP). http://www.tutiempo.net/clima. Arrows indicate sampling times.

**Figure 3:**
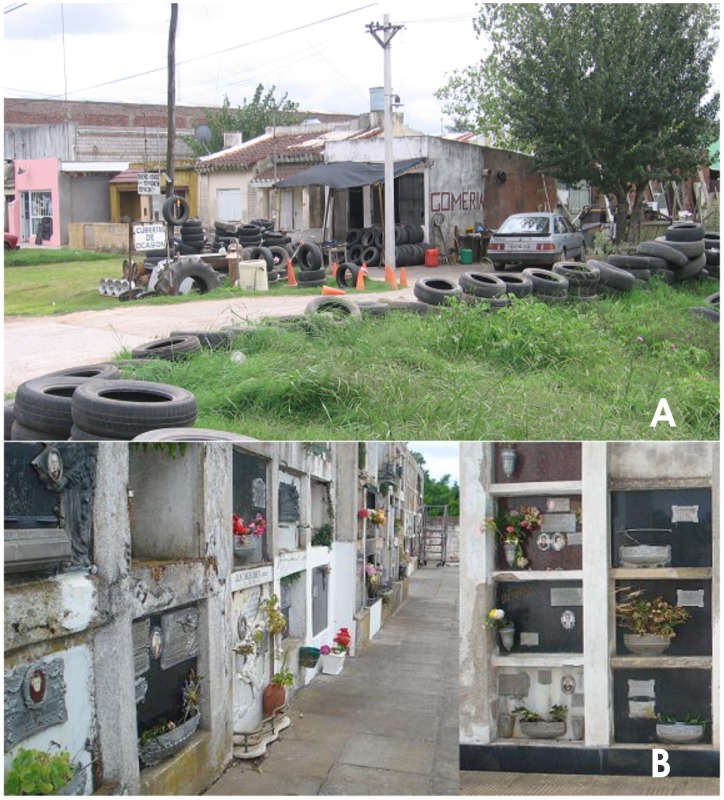
Sampling locations in Buenos Aires province. (**A**) Tire-repair stations showing tires with accumulated rainwater. (**B**) Flowerpots at cemeteries.

One of the most important highways in Argentina is Provincial Route N°2, which connects Buenos Aires and La Plata cities with Mar del Plata city and the most visited beaches of the country, principally in the summer time, representing about 2 million people commuting between those places (Figure 1C and Table 1) (http://www.indec.mecon.ar). Route N°2 crosses the most prominent wetland areas of the Pampas, and its construction has definitely reshaped the landscape, making available new humanmanmade wetlands that offer shelter to an increasing diversity of flora and fauna, including mosquitoes [19]. On this artery there are some small towns that offer several travel services such as tire-repair stations, or “gomerías”, which store used automobile and truck tires for long periods of time; thus these tires accumulate rainwater (Figures 2 and 3A). Moreover, along this highway many vehicles transport goods from the north of the country to the coastal area without any sanitary control to prevent insect exchange from one region to the other. The latest scientific southernmost record of *A. aegypti* detected in Buenos Aires Province was obtained in Chascomús, a town located on Route N°2 [11]. Route N°2 takes the bulk of the traffic and people in a southeastern direction. On the other hand, Route N°11, connecting Buenos Aires and La Plata cities with the Atlantic coast, is a short motorway parallel to the coastline, and Route N°226 runs southwest and is mostly used by freight transport (Figure 1C).

## Present Distribution of *A. aegypti* in the Most Populated Areas of Buenos Aires Province

In order to understand the status of the southern distribution of *A. aegypti*, we sampled mosquito larvae and pupae during the rainy period, in January and March 2011, and only in March 2012, because rainfall levels were very low in January 2012 (Figure 2). The sampling stations were located in towns situated along Route N°2 and the other two major arteries that connect Buenos Aires with the south. The sampling stations were cemeteries that are far from the towns and are infrequently visited, and gomerías located in densely populated areas of each town, both at the edge of the roads (Figure 3 and Table 2). Larval specimens were collected and reared until fourth instar or adult stage to facilitate identification using specific keys [13], [20]. Voucher specimens, prepared from all localities, were submitted to the local museum, Museo de Ciencias Naturales “Lorenzo Scaglia” (Mar del Plata, Argentina).

**Table 2:** Sampling stations and species collected in cities along Route N° 2, in the southeast of Argentina.

City	2011						2012					
	Flowerpots^a^	*Culex* sp.	*A. aegypti*	Tire-Repair Stations^b^	*Culex* sp.	*A. aegypti*	Flowerpots^a^	*Culex* sp.	*A. aegypti*	Tire-Repair Stations^b^	*Culex* sp.	*A. aegypti*
**Chascomús**	239 (12/0)	+	−	1 (1/1)	+	+	300 (8/2)	+	+	2 (2/2)	+	+
**Lezama**	200 (0/0)	−	−	3 (3/1)	+	+	200 (5/0)	+	−	3 (3/3)	+	+
**Castelli**	480 (0/0)	−	−	3 (3/0)	+	−	200 (3/2)	+	+	3 (3/3)	+	+
**Dolores**	730 (29/0)	+	−	2 (2/0)	+	−	400 (12/1)	+	+	2 (2/2)	+	+
**Gral. Guido**	280 (1/0)	+	−	2 (2/0)	+	−	300 (7/0)	+	−	3 (3/0)	+	−
**Maipú**	440 (5/0)	+	−	2 (2/0)	+	−	nd^c^	nd^c^	nd^c^	2 (1/0)	+	−
**Mar del Plata**	3,600 (∼45/0)	+	−	10 (8/0)	+	−	3,600 (∼45/0)	+	−	10 (8/0)	+	−

a Number of flowerpots sampled, in brackets positive ones for *Culex* sp. and for *A. aegypti*, respectively.

b The number of *A. aegypti* was 500 larvae or more in each tire-repair station, in brackets positive ones for *Culex* sp. and for *A. aegypti*, respectively.

c No data.

Larvae of *A. aegypti* were found in March 2011 and 2012 in Chascomús, agreeing with and confirming previous records [11], [13]. Here we report the finding of *A. aegypti* in the towns of Lezama, Castelli, and Dolores, to our knowledge for the first time. A population of mosquitoes was found in Lezama in March 2011, 39.2 km southeast of Chascomús; both localities are separated by farmland and uniquely connected by Route N°2. As a high number of larvae of all stages and pupae were found in multiple containers in Lezama, we feel confident that this locality holds a natural, well-established population. In March 2012, we found a higher number of larvae of all stages and pupae in the same type of containers for a second time in Lezama, and for the first time in Castelli (27.7 km south from Lezama) and Dolores (59.5 km south from Lezama), making Dolores the southernmost limit of the species' range within Argentina, now 98.7 km south of Chascomús (Figure 1C). In Routes N°11 and 226, *A. aegypti* was not found in any of the water containers examined.

In the south of Argentina, *A. aegypti* is very likely to be moving by passive dispersal using the major highway connecting the north with the southeast of the country. It is noteworthy that this same behavior has been studied and documented in a closely related species *A. albopictus* in the US. Previous observations on this mosquito in North America are consistent with the hypothesis of mosquito migration facilitated by anthropic action, presumably by transportation of scrapped tires through the interstate highway system [17]. In *A. aegypti*, egg resistance in the absence of water, a feature shared with *A. albopictus*, can lead to a similar way of transferring to new places in order to breed. Therefore, passive dispersal of *Aedes* species using frequented freeways should be considered when designing new *A. aegypti* monitoring programs.

According to Shepherd et al. [21], dengue virus transmission follows two general patterns: epidemic dengue and hyperendemic dengue. Epidemic dengue transmission occurs when dengue virus is introduced into a region as an isolated event that involves a single viral strain. If the number of vectors and susceptible hosts are sufficient, explosive transmission can occur with an infection incidence of 25%–50%. Hyperendemic dengue transmission is characterized by the circulation of multiple viral serotypes in an area with susceptible hosts and competent vector (with or without seasonal variation) and appears to be a major risk for dengue hemorrhagic fever. Travelers to these areas are more likely to be infected than travelers going to areas that experience only epidemic transmission.

In South America, particularly in Buenos Aires Province, it is known that the provincial health ministry has a program of surveillance of *A. aegypti*, which involves the monitoring and control of mosquito larvae and eggs. However, this surveillance does not follow a regular pattern, being erratic in terms of time, and each council or municipality decides whether to carry it on or not. In addition, sometimes it is difficult to obtain official data.

The new biogeographical record of central and southern Argentina, reported in this article, is an important fact of the constant expansion of *A. aegypti* into new southernmost areas. Together with the presence of the different dengue serotypes, this expansion indicates that the situation is far more dangerous than previously thought. Urgent and responsible actions must be taken to control the dengue vector and its expansion into new areas.
